# Clinicopathological Parameters of Haemophilia Patients at a Tertiary Care Centre in Northern India

**DOI:** 10.7759/cureus.41670

**Published:** 2023-07-10

**Authors:** Anurag Singh, Shalini Rawat, Rashmi Kushwaha, Mili Jain, Shailendra P Verma, U.S. Singh

**Affiliations:** 1 Pathology, King George's Medical University, Lucknow, IND; 2 Clinical Hematology, King George's Medical University, Lucknow, IND

**Keywords:** epistaxis, ecchymosis, post-traumatic bleed, joint bleed, hemophilia

## Abstract

Introduction: Haemophilia affects a large number of people all over the world, yet very little is known about the clinical manifestations and diagnostic protocols of the condition in areas with limited access to resources in developing countries. Understanding the clinical spectrum and diagnostic approach will help with the design of measures to address the situation in these places. The primary objective of this study was to examine the clinicopathological parameters of haemophiliac patients.

Materials and methods: From the departmental archive, a thorough history of each patient was retrieved, including values of bleeding time, prothrombin time, activated partial prothrombin time, and percentage of specific factor activity.

Results: Out of a total of 385 cases over the period of six years, 86.75% were classified as haemophilia A and 13.25% of cases were diagnosed as haemophilia B. In terms of the severity of the disease, 44.93% were classified as severe, 42.08% as moderate, and 12.99% as mild. Joint bleeding was the first and most typical clinical manifestation of the disease, accounting for 34.80% of cases, followed by ecchymosis (23.12%), post-traumatic bleeding (12.73%), epistaxis (12.20%), and gum bleeding (8.05%). 1.56% of patients had a positive screening test for the hepatitis C virus, followed by 1.30% for HIV and 0.78% for hepatitis B surface antigen.

Conclusion: In the presence of joint bleeding, ecchymosis, and post-traumatic bleeding in an otherwise healthy individual, a clinician should be alerted to the possibility that the patient has haemophilia and should request a work-up for the bleeding disorder.

## Introduction

The most prevalent congenital coagulation factor deficiencies, haemophilia A (factor VIII [FVIII] deficiency) and haemophilia B (factor IX deficiency), are characterised by protracted and profuse bleeding after minimal trauma or occasionally even spontaneously. Worldwide, haemophilia affects all racial and ethnic groups [1]. In the overall haemophilia population, haemophilia A predominates over haemophilia B (80% to 85%). It manifests in one in 5,000 live male births, compared to one in 30,000 live male births for haemophilia B [2]. Ecchymosis, epistaxis, post-traumatic bleeding, gastrointestinal bleeding, haemarthrosis, haematomas, and central nervous system (CNS) bleeding are only a few of the clinical manifestations of the illness. The percentage of residual FVIII coagulant activity (FVIII:C) is closely correlated with the severity of haemophilia A. Joint and muscle bleeding, which typically start when the child begins to walk, are the most prevalent haemophilia symptoms. Patients with haemophilia are classified based on the levels of endogenous residual FVIII/FIX. Normal levels of FVIII range from 50% to 150% [3]. Subjects having factor levels of less than 1 IU/dL, between 1 and 5 IU/dL, and greater than 5 IU/dL, respectively, have severe, moderate, and mild haemophilia [3,4]. Haemophilia necessitates early clinical suspicion, specialized tests for diagnosis, and lifetime support, which places a burden due to the huge population in the northern area of India. The purpose of this study was to fill the information gap by evaluating the clinicopathological profile of haemophilia in patients who presented to this tertiary care institution at the time of diagnosis.

## Materials and methods

This is a six-year (January 2017 to December 2022) retrospective descriptive study carried out at the Department of Pathology, King George Medical University Lucknow, Uttar Pradesh, a tertiary care centre in North India. From the departmental archive, a thorough history of each patient was retrieved, including the age of diagnosis, family history, bleeding manifestations, joint involvement, epistaxis, bleeding gums, haematuria, blood in the stool or vomit, medication history, blood loss during surgical, circumcision, or dental procedures, and parental consanguinity at the time of the initial diagnosis.

Participants who were on anticoagulant therapy or antiplatelet medications, had active bleeding from trauma with bleeding disorder other than haemophilia, a history of snakebite within the previous month, and critically ill patients who were unable to provide informed consent were excluded from the study. For the coagulation profile, a 3.2% sodium citrate vacutainer was employed as an anticoagulant in a 1:9 ratio and processed over the course of 4 hours. To obtain complete blood counts, platelet counts, and a peripheral blood smear examination, ethylene diamine tetraacetate (EDTA) blood samples were employed. For coagulation studies, the tests were conducted on platelet-poor plasma. Patients' coagulation profiles were determined using prothrombin time (PT), activated partial thromboplastin time (aPTT), thrombin time, and correction tests using pooled plasma, normal aged serum, and Al(OH)3 adsorbed plasma. The modified Ivy's approach was used for bleeding time (BT). On an automated coagulometer (STA Compact Max), a specific factor assay for factors VIII and IX was performed. The departmental archive was used to record the BT, PT, aPTT, and residual factor activity values.

The statistical method used frequency and percentages to convey a descriptive analysis of quantitative data. The statistical average used was the mean value, and the standard deviation was used to calculate dispersion.

## Results

There were 385 cases of haemophilia included in the current study. Haemophilia is categorised into three different groups based on factor activity: mild (>5 to 40%), n = 50 (12.99%); moderate (1-5%), n = 162 (42.08%); and severe (1%), n = 173 (44.93%) (Table 1).

**Table 1:** Classification of haemophilia cases according to their severity

	Factor levels	Number of cases (n)	Percentage of cases (%)
Severe	<1%	173	44.93
Moderate	1-5%	162	42.08
Mild	>5-40%	50	12.99
	Total	385	100

Haemophilia A affected 86.75% (334/385) of patients and haemophilia B affected 13.25% (51/385). The mean age of the patients in the study was 11.75 ± 3.12 years, and 32.47% (125/385) of the cases were over the age of 15 years. 72.73% (280/385) participants belonged to the Hindu religion, 27.01% (104/385) belonged to the Muslim religion, and 0.26% (1/385) belonged to the Christian community. Two hundred and twenty-six (58.70%) patients were from rural areas and 159 (41.30%) were from urban areas. A positive family history was identified in 40.52% (156/385) of the cases. Fifty-four percent (27/50) of cases in the mild haemophiliac group had age >15 years, followed by 32.72% (53/162) of cases among moderate haemophiliacs and 26.01% (45/173) of cases with severe disease. The lowest percentage, 2% (1/50), of cases with age <1 year belonged to the mild haemophiliac group, with an increasing percentage among moderate haemophiliacs of 5.56% (9/162) and severe disease of 11.56% (20/173). There was a statistically significant difference noted for age (p = 0.001) between the severe, moderate, and mild groups of haemophiliac patients (Table 2). At the time of their first bleeding symptom, 52.47% (202/385) of the patients were diagnosed with haemophilia.

**Table 2:** Association of demographic profile with the severity of haemophilia

		Severe (n = 173)		Moderate (n = 162)		Mild (n = 50)		p-Value
		n	%	n	%	n	%	
Age at the time of diagnosis	<1 year	20	11.56	9	5.56	1	2.00	0.001
	1-5 years	52	30.06	49	30.25	8	16.00	
	6-10 years	31	17.92	29	17.90	13	26.00	
	11-15 years	25	14.45	22	13.58	1	2.00	
	>15 years	45	26.01	53	32.72	27	54.00	
Religion	Hindu	130	75.14	123	75.93	27	54.00	0.020
	Muslim	42	24.28	39	24.07	23	46.00	
	Christian	1	0.58	0	0.00	0	0.00	
Residence	Rural	106	61.27	97	59.88	23	46.00	0.143
	Urban	67	38.73	65	40.12	27	54.00	

The first line of investigations in the laboratory examination of inherited bleeding diseases included complete blood counts, peripheral blood smears, platelet counts with platelet morphology, clot retraction (CR), BT, PT, and aPTT. The platelet counts, BT, and PT of each patient in the current study were within normal ranges. All haemophilia cases had increased aPTT. The increasing values of aPTT significantly correlated with illness severity (p < 0.001) (Table 3).

**Table 3:** Association of mean bleeding time, prothrombin time, and activated partial prothrombin time with the severity of haemophilia

	Severe (n = 173)		Moderate (n = 162)		Mild (n = 50)		p-Value
	Mean	±SD	Mean	±SD	Mean	±SD	
BT (seconds)	178.86	65.58	176.27	53.74	174.40	46.20	0.863
PT (seconds)	13.97	1.18	14.16	4.60	13.84	1.02	0.761
aPTT (seconds)	84.56	23.76	63.68	15.02	52.64	11.38	<0.001

**Figure 1:**
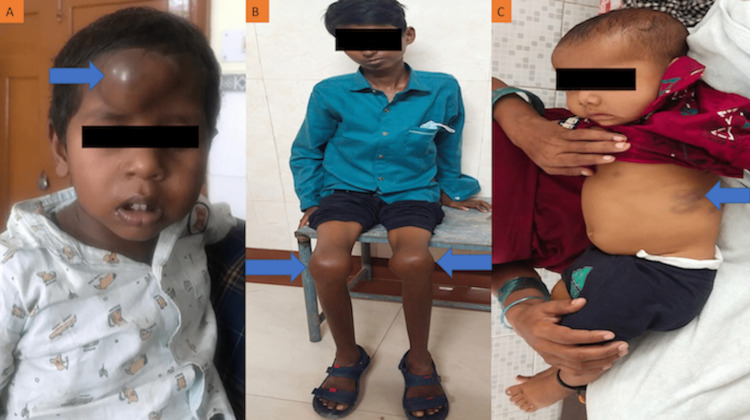
Spectrum of clinical manifestations in haemophilia patients (A) A five-year-old child displaying extradural haematoma over the forehead. (B) A 13-year-old child showing bilateral knee involvement by the disease. (C) A three-year-old child displaying a large ecchymosis patch over the left lateral aspect of the abdomen.

**Figure 2:**
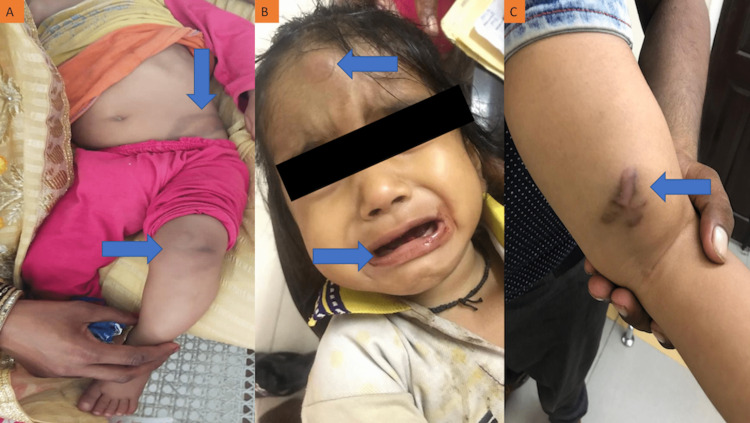
Spectrum of clinical manifestations in haemophilia patients (A) A two-year-old child showing left-side knee involvement and ecchymosis patch over the left flank. (B) A nine-month-old child displaying extradural haematoma over the forehead and gum bleed. (C) A one-year-old child displaying an ecchymosis patch over the posterior aspect of the thigh.

Knee joint bleeding was presented as their first clinical manifestation by 24.86% (43/173) of the severe haemophilic patients, followed by ecchymosis (23.0%, 40/173), gum bleeding (12.72%, 22/173), and epistaxis (11.56%, 20/173). Among moderate haemophilic patients, the first clinical manifestation was ecchymosis (27.16%, 44/162), followed by knee joint bleeding (20.99%, 34/162), epistaxis (14.81%, 24/162), and post-traumatic bleeding (11.73%, 19/162). Fifty-four percent (27/50) of the mild haemophilic patients presented post-traumatic bleeding as their first clinical manifestation, followed by post-surgical bleeding (16.0%, 8/50), ecchymosis (10.0%, 5/50), and epistaxis (6%, 3/50) (Figures 1, 2).

The knee was the most common target joint involved in haemophilic patients (20.78%, 80/385), followed by the ankle joint (6.49%, 25/385), elbow joint (3.12%, 12/385), hip joint (2.60%, 10/385), and shoulder joint (1.30%, 5/385). For moderate haemophiliacs, ankle joint bleeding (9.26%) and shoulder joint bleeding (3.09%) were the two most frequent initial presentations, while bleeding in the knee joint (24.86%), hip joint (4.62%), and elbow joint (4.05%) was more prevalent among severe haemophiliacs. Intracranial bleeding (1.73%), bleeding in the proximal metacarpophalangeal (MCP) joint (0.58%), and bleeding in the wrist joint (0.58%) occurred only in severe haemophiliac cases (Table 4).

**Table 4:** Correlation between first clinical manifestations of haemophilia cases and disease severity MCP, metacarpophalangeal.

	Severe (n = 173)		Moderate (n = 162)		Mild (n = 50)		Total
	n	%	n	%	n	%	
Ankle joint bleeding	10	5.78	15	9.26	0	0.00	25
Ecchymosis	40	23.12	44	27.16	5	10.00	89
Elbow joint bleeding	7	4.05	5	3.09	0	0.00	12
Epistaxis	20	11.56	24	14.81	3	6.00	47
Gastrointestinal bleeding	4	2.31	0	0.00	2	4.00	6
Gum bleeding	22	12.72	8	4.94	1	2.00	31
Haematuria	11	6.36	4	2.47	1	2.00	16
Hip joint bleeding	8	4.62	2	1.23	0	0.00	10
Intracranial bleeding	3	1.73	0	0.00	0	0.00	3
Knee joint bleeding	43	24.86	34	20.99	3	6.00	80
Post-traumatic bleeding	3	1.73	19	11.73	27	54.00	49
Post-surgery bleeding	0	0.00	1	0.62	8	16.00	9
Proximal MCP joint bleeding	1	0.58	0	0.00	0	0.00	1
Shoulder joint bleeding	0	0.00	5	3.09	0	0.00	5
Wrist joint bleeding	1	0.58	0	0.00	0	0.00	1

At our institute, the initial symptomatic treatment was given to each and every haemophilia patient. Cryoprecipitate was administered to 54.29% (209/385) of the patients, fresh frozen plasma was given to 29.09% (112/385), and clotting factors were given to 36.62% (141/385). In 22.5% (87/385) of the cases, the patient received a whole blood transfusion. 1.56% (6/385) of patients had a positive screening test for the hepatitis C virus. This was followed by 1.30% (5/385) for HIV and 0.78% (3/385) for hepatitis B surface antigen-positive transfusion-transmitted infection.

## Discussion

A major public health issue, coagulopathy causes increased mortality and morbidity as a result of spontaneous or traumatic bleeding events. Haemophilia, von Willebrand disease, and thrombocytopenia are the three main causes of coagulopathy [4]. FVIII or factor IX deficiency is an X-linked inherited bleeding condition known as haemophilia A and B, respectively. A lack of these components may cause repeated bleeding into muscles and joints, resulting in haemophilic arthropathy and contractures [5]. Developing nations like India are thought to be home to 80% of the world's haemophiliacs because of a lack of social awareness about inherited bleeding disorders [6]. We found 385 cases of haemophilia in our study; 86.75% were haemophilia A and 13.25% were haemophilia B. These results are in concordance with previous studies [2,7,8].

44.93% (173/385) of the haemophilia patients have severe disease, followed by moderate haemophilia (42.08%, 162/385), and mild in 12.99% (50/385) of cases. An increased prevalence of haemophilia with severe disease was also noted in earlier Indian research [7,8]. The studies from Western countries found a larger percentage of mild cases in their study compared to moderate and severe diseases [9,10]. The reported factor activity in haemophiliac patients among different racial populations may differ due to geographic distribution, disease awareness, or variability in clinical manifestation, among other possible multifactorial causes.

Because haemophilia is a hereditary condition, family history has been documented in 40-70% of cases [11,12]. According to the findings of our study, 40.52% of cases had a positive family history.

Patients with haemophilia have a wide range of clinical symptoms, and even those with severe disease exhibit a variety of bleeding patterns [13]. The haemophilia classification renders direction to different potential types of bleeding manifestations that may present at varying ages depending on severity. Patients with a severe form of haemophilia bleed spontaneously, whereas those with a moderate form bleed after minor to moderate injuries. Patients with mild haemophilia may go years without being diagnosed and appear with bleeding after surgery or trauma [14]. As in the current study, the group of mild haemophiliacs accounted for the highest percentage of cases with an age >15 years, followed by the group of moderate and severe haemophiliacs.

Haemarthrosis, or bleeding into a joint space, is the most typical sign of haemophilia. Large joints, usually the knee and ankle joints, are engaged in serious disease at varying frequencies; the results of our study also supported these findings [15-17]. The first presenting clinical manifestation of bleeding episodes among severe, moderate, and mild haemophilia patients is described in the current study.

The most frequent presenting characteristic was identified as joint bleeding (34.8%) in the present study, followed by skin bleeding (23.12%). In Jodhpur, India, Payal et al. also reported skin bleeds and haemarthrosis as the most common clinical manifestations in haemophiliac patients in their study [15]. The present study showed that the knee joint is the most afflicted, followed by the ankle joint, elbow joint, hip joint, and shoulder joint. The findings of the current study are consistent with those found by Karim et al. and Mohsin et al. in their study carried out in a tertiary care hospital [16,17]. Among haemophiliacs, intracranial haemorrhage is a leading cause of morbidity and mortality and is seen in 3-4% of severe cases [18]. In the current study, 1.73% (3/173) of the severe haemophilia cases exhibited cerebral bleeding, which is a few percentage points less than in the prior studies. In comparison to individuals with severe haemophilia (23.12%), moderate haemophilia patients (27.16%) had more bruises and ecchymosis in the present study.

Additionally, we found that nearly one-third of cases had delayed diagnoses (>15 years), which indicates that patients from developing countries are less likely to be aware of their condition when it first manifests, particularly in cases with mild disease. This demonstrates that there is still a knowledge gap regarding disease awareness among patients, clinical suspicion, and diagnosis in cases of haemophilia. In the case of underdeveloped nations, the diagnostic evaluation of suspected cases of haemophilia may frequently be hampered by a lack of facilities to measure FVIII, factor IX, FVIII inhibitor, and von Willebrand factor levels. As a result of the bleeding manifestation occurring at an older age, the diagnosis is more difficult in neglected cases of mild to moderate severity [19]. A haemophilia campaign must be launched among developing nations to raise awareness, and a rigorous, cautious approach to evaluating bleeding symptoms is necessary to make a conclusive diagnosis of haemophilia.

Patients suffering from haemophilia continue to face a significant obstacle in the form of transfusion-transmitted infections. Hepatitis C virus infection remains the most significant source of worry for haemophiliacs; however, a few patients were also positive for hepatitis B and HIV. Patients with haemophilia should get vaccinated against hepatitis B as soon as possible [20,21]. This study had few limitations because it was retrospective and conducted at a single centre. Additionally, the sample size of patients was small, and they were further divided into groups based on the severity of the disease.

## Conclusions

To conclude, patients with haemophilia can have a broad range of clinical manifestations and may need ongoing treatment. Haematopathologists play a crucial role in the timely detection of bleeding disorders through the analysis of clinical presentation, basic coagulation screening, and diagnostic testing. An early diagnosis of haemophilia plays a critical role in the preventative prescription of clotting factors, which in turn leads to a reduction of anaemia, transfusion-transmitted infections, and joint haematoma. 

## References

[1] Peyvandi F, Garagiola I, Young G (2016). The past and future of haemophilia: diagnosis, treatments, and its complications. Lancet.

[2] Kar A, Phadnis S, Dharmarajan S, Nakade J (2014). Epidemiology & social costs of haemophilia in India. Indian J Med Res.

[3] Pishko AM, Doshi BS (2022). Acquired hemophilia A: current guidance and experience from clinical practice. J Blood Med.

[4] Aynalem M, Shiferaw E, Gelaw Y, Enawgaw B (2021). Coagulopathy and its associated factors among patients with a bleeding diathesis at the University of Gondar Specialized Referral Hospital, Northwest Ethiopia. Thromb J.

[5] Gualtierotti R, Solimeno LP, Peyvandi F (2021). Hemophilic arthropathy: current knowledge and future perspectives. J Thromb Haemost.

[6] Ghosh K, Ghosh K (2016). Management of haemophilia in developing countries: challenges and options. Indian J Hematol Blood Transfus.

[7] Kumar S, Sinha S, Bharti A, Meena LP, Gupta V, Shukla J (2019). A study to determine the prevalence, clinical profile and incidence of formation of inhibitors in patients of hemophilia in North Eastern part of India. J Family Med Prim Care.

[8] John MJ, Tanuja T, Mathew A (2018). Demographic profile and real world data of persons with hemophilia in a resource constrained setup. CHRISMED J Health Res.

[9] Aznar JA, Lucía F, Abad-Franch L (2009). Haemophilia in Spain. Haemophilia.

[10] Hart DP, Matino D, Astermark J (2022). International consensus recommendations on the management of people with haemophilia B. Ther Adv Hematol.

[11] Thomas W, Downes K, Desborough MJ (2020). Bleeding of unknown cause and unclassified bleeding disorders; diagnosis, pathophysiology and management. Haemophilia.

[12] Öner N, Gürsel T, Kaya Z (2020). Inherited coagulation disorders in Turkish children: a retrospective, single-center cohort study. Transfus Apher Sci.

[13] Coppola A, Di Capua M, Di Minno MN (2010). Treatment of hemophilia: a review of current advances and ongoing issues. J Blood Med.

[14] van Dijk K, Fischer K, van der Bom JG, Grobbee DE, van den Berg HM (2005). Variability in clinical phenotype of severe haemophilia: the role of the first joint bleed. Haemophilia.

[15] Payal V, Sharma P, Goyal V, Jora R, Parakh M, Payal D (2016). Clinical profile of hemophilia patients in Jodhpur Region. Asian J Transfus Sci.

[16] Karim MA, Siddique R, Jamal CY, Islam A (2013). Clinical profile of hemophilia in children in a tertiary care hospital. Bangladesh J Child Health.

[17] Mohsin S, Saeed T, Hussain S, Mahmood S, Sohail S (2010). Clinical manifestations and complications of hemophilia A in Pakistan. Ann Pak Inst Med Sci.

[18] Ljung R, Chambost H, Stain AM, DiMichele D (2008). Haemophilia in the first years of life. Haemophilia.

[19] Islam MN, Biswas AR, Nazneen H (2022). Clinical profile and demographic characteristics of moderate and severe hemophilia patients in a tertiary care hospital of Bangladesh. Orphanet J Rare Dis.

[20] Borhany M, Shamsi T, Boota S (2011). Transfusion transmitted infections in patients with hemophilia of Karachi, Pakistan. Clin Appl Thromb Hemost.

[21] Peng HM, Wang LC, Zhai JL, Weng XS, Fen B, Wang W (2020). Transfusion-transmitted infections in hemophilia patients who underwent surgical treatment: a study from a single center in north China. Arch Med Sci.

